# The Influence of the Mental over Man's Physical Forces

**Published:** 1872-12

**Authors:** A. B. Jones


### SELECTED ARTICLES.

---

### ARTICLE V.

### *The Influence of the Mental over Man's Physical Forces.*

BY A. B. JONES, M. D.

We prick the finger with a needle, and instantaneously a nerve of sensation gives us an intelligent idea of violence. By the aid of the scalpel and microscope we are enabled to follow up the nerve fibre to its starting point. No sooner has the sensation reached what we are taught to call the root of the mind, the brain, than another set of nerve fibres spring into action, and at once withdraw the finger from further injury. These nerves of sensation and motion are simply prolongations of the medullary substance of the brain, spinal cord and semi-lunar ganglions, which find their way to every part of the body. Had no other office been assigned to the brain than the control of these forces alone, it would have a duty of highest importance to perform. But we have an office assigned it infinitely higher than that of sensation or motion. The brain is the workshop of man's mental forces; this he will neither assume nor deny, holding either position beyond demonstration, but simply ask, where are the nerves of thought? Are they wrapped up in the gray or white substance of the brain, to grow and strengthen as Prof. Agassiz advises us, by eating fish? or as Mark Twain pertinently suggests, a whale? Or are they lying loose in the front and large portion of the brain, the one-fourth of which I have seen a boy lose, from a fracture caused by a gunshot wound, without affecting his mind? But the object of this paper is not so much to hunt up the exact location of man's mental forces, as to show their influence upon physical organs, the exact location of which we do understand, together with their functions. That a man may enjoy good physical health with a very feeble intellect is a fact so well established that but few will controvert it.

But the converse will not hold good; the immortal part of man, that which was made in the likeness and image of the Infinite, is dependent for its proper, full and vigorous development upon the healthy and well-developed condition of all the physical organs.

A man is no more capable of reasoning correctly who is in confirmed hypochondria, which we have been taught to believe has its origin often in the derangements of digestion, than he would be with softening of the brain, or, if you please, with a tumor on the brain. Yet the mind, so to speak, may dwarf the man physically to such an extent as to cut off its own supply; and it is this, as guardians of the health and preservation of our race, that we are daily called upon to consider, and, it may be, to correct. The influence of the mind is more frequently, and, perhaps, more directly, felt upon the stomach than any other organ. Who of us has not sat down to dinner with a keen relish for the good things set before us, when some sudden news, depressing, perhaps, in its character, has in a moment induced satiety. Intense grief or fear is said to have changed the color of the hair in a single night from black to white. An over anxious feeling, coupled with hope and doubt, is very apt to increase the secretion of the kidneys. Prurient thoughts will increase the secretion of semen; the cry of a young child will start the lacteal flow in the mother; the fear and dread of a cancer have, without much doubt, converted a simple fibrinous tumor of the breast into a malignant one; putrid or disgusting objects may produce emesis; and there is but little doubt but that the mental emotions may be so operated upon as to cause an attack of diarrhœa. And so we might go on until we had enumerated nearly all of the secreting organs of the body. Men, in good health, meet with some little reverse in their business; they grow anxious about it, lose sleep and appetite, then they worry because they cannot either eat or sleep, until they become sick. The physician is told everything but the truth, when he proceeds to worry them additionally with drugs. When death supervenes, they die of "softening of the brain," a very convenient disease for men to die of; the skull is so thick that you cannot conveniently feel the brain through it; and then if an autopsy is made, why, the brain is always sure to be soft, which proves eminently satisfactory to the friends.

A sick person grasps the thoughts of a physician the moment he enters the chamber, and he holds them as if they were things tangible, just as he does the outstretched hand, only he holds them long after the doctor has gone on his weary way. Hence the quiet, cool, easy, cheerful, self-possessed, confident doctor, is always the successful practitioner. A physician once wished to compliment a lady, who had brought a floral tribute to one of his sick patients, and while looking upon the beautiful bouquet of flowers close to the blanched cheek, he politely remarked that he once knew life and death poised in the balance, and the delicate odor of the citron turned the scale in favor of life. While this may not be literally true, it is not without its effect, and it is as much the duty of a physician to look after and control that spirit essence, or subtle essence, the mind, as it is to know that the stomach has been relieved of its noxious bile, or that the fevered pulse now keeps pace with his own. The finest medical lecture ever given, at least in so short a compass, was by Solomon ; it is this : "A merry heart doeth good like a medicine, but sorrow is as rottenness in the bones."

It is a common saying that you must have faith in a doctor, or his medicine will not cure you. Now confidence and trust in a physician's skill is, no doubt, oftentimes fraught with good results. The solution of this is that the mind is relieved, in a measure, of its anxiety, ceases to concentrate itself upon the diseased organ ; but this is not so much a cure by faith as it is a cure by mental over physical force. It is not only that one's own physical organs are influenced by mental force, or mesmeric force, but that through the latter, or nerve force, one's mind exercises a material influence over another's physical organs. This pertinently suggests the necessity of a thorough study and knowledge of human nature, by the well-educated physician. It is not unfrequently the case that we find physicians eminently qualified to practice their profession who are very unsuccessful in their practice. Why is this ? It is certainly not because their diagnosis has been faulty, neither is it because improper medicines have been used ; but it is because the mind of the patient in stretching forth its delicate tendrils—may be bruised ones—found nothing to refresh and strengthen them. Simply because the souls of the physician and patient, or their minds, did not seek to get acquainted with each other. This certainly is the chief cause of their failure, and tells us very plainly that our minds, our souls, our thoughts, must be administered, as well as our drops and pills, if we would successfully combat disease.

We occasionally meet persons with imaginary diseases—imaginary in the beginning, but real in the ending. Again, there are some persons who have had real disease, been thoroughly cured, and yet their minds, so to speak, remain so full of the disease that they cannot be made to believe they are well, and a depressing influence is thus brought to bear upon their general health, closely allied, if not akin, to the trouble they have been cured of. Now, blister, plaster and quinine will not relieve this class of patients; they can only be cured by administering to what we have been taught to call a diseased mind an equal amount of healthy mind.

How is it to be done? Well, there comes the rub; it is enough for my present purpose to say it must be done. Association occasionally develops disease, for instance, chorea. A boarding-school miss gets sick, recovers in a degree, but there remains with her an involuntary motion of some of her limbs, beyond her control. This occasionally extends through an entire class, almost as much so as rubeola or pertussis would. This, of course, I do not class as a disease of the mind, but rather as one that may be caused by acting through the mind. One other class of patients and I am done. A patient comes to you, a highly intelligent gentleman, a lawyer perhaps, may be a divine; he has read a great deal, he has thought a great deal, and no doubt but he knows a great deal in his line. He holds in his hand an advertisement, which he has cut from a newspaper, of some patent medicine man, or it may be a leaf out of Jayne's almanac; possibly some disciple of Hahnemann has sugared him up by his description of the aches and ills that flesh is heir to. Now, he wishes you to distinctly understand that he does not believe in patent medicines, and as for those little pills, he thinks nothing could be more insignificant, unless, perhaps, it might be the knave or ass who peddles them. "But then," continues he, "they have described my feelings better than I could do it myself, and it may be possible that this is just what I need." You examine the case carefully, and find instead of his needing a "Liver Invigorator," "Lung Balsam," "Blood Purifier," or "Catarrh Snuff," that he has simply overtaxed himself, both mentally and physically, until he can easily imagine aches he does not feel. But there is still another, and perhaps better reason; it is this: nearly every patent medicine man describes about the same class of symptoms, in about the same words, no difference what the disease he is describing. This is not noticed by the general reader. It requires only a little careful wording, with a moderate degree of ingenuity, to tell a man about how he feels, for there is scarcely any sick man who feels well. A little address may be well in the beginning; for instance, preface your remarks with, "You know from your own personal observation that he is a man who will not give up to trifles; that he has a general feeling of languor and debility all over; an occasional chilliness, followed with more or less fever, flashes of heat, a general aching all over, an occasional palpitation of the heart, a little nervous, will start suddenly if frightened, irregular appetite, cannot sleep well, a little running round of the head if he stoops down and rises up suddenly; after eating a hearty meal he gets up from the table feeling full." Now, this will satisfy nine men out of ten. Of course, I need not here remark that this is all absurd, but nevertheless it is kindred stuff, through patent medicine advertising, that causes a great deal of the diseases of both the mind and body that we are called upon to treat, and I merely refer to it to illustrate the action of the forces of which this paper is the subject.—*Med. and Surg. Rep.*

---

